# What is empathy for?

**DOI:** 10.1007/s11229-015-0771-8

**Published:** 2015-05-30

**Authors:** Joel Smith

**Affiliations:** School of Social Sciences, University of Manchester, Manchester, M13 9PL UK

**Keywords:** Empathy, Fellow-feeling, Knowing what it’s like

## Abstract

The concept of empathy has received much attention from philosophers and also from both cognitive and social psychologists. It has, however, been given widely conflicting definitions, with some taking it primarily as an epistemological notion and others as a social one. Recently, empathy has been closely associated with the simulationist approach to social cognition and, as such, it might be thought that the concept’s utility stands or falls with that of simulation itself. I suggest that this is a mistake. Approaching the question of what empathy is via the question of what it is for, I claim that empathy plays a distinctive epistemological role: it alone allows us to know how others feel. This is independent of the plausibility of simulationism more generally. With this in view I propose an inclusive definition of empathy, one likely consequence of which is that empathy is not a natural kind. It follows that, *pace* a number of empathy researchers, certain experimental paradigms tell us not about the nature of empathy but about certain ways in which empathy can be achieved. I end by briefly speculating that empathy, so conceived, may also play a distinctive social role, enabling what I term ‘transparent fellow-feeling’.

## Introduction

It is a commonplace to point out that while research on empathy is burgeoning, there is little agreement amongst empathy researchers about what it is (Batson 2009; Goldman 2011). Candidates, crudely described, include our automatic and often non-conscious imitation of others’ facial expressions, vocal expressions and posture (Van Baaren et al. 2009); our ‘catching’ of, ‘mirroring’, or ‘resonating’ with, other people’s affective states—emotional contagion—that is sometimes claimed to ensue from such imitation (Rapson et al. 1994; Hatfield et al. 2009); our knowledge of the source of such imitation or contagion in another subject (De Vignemont and Singer 2006); our imagining another subject’s situation, either as ourselves or as them (imagine-self vs. imagine-other perspective-taking) (Batson et al. 1997; Goldie 2011); or our feeling as the other does as a result of such an imaginative project (Coplan 2011). In addition, there are a number of accounts that build in some element of concern for the other (Batson 2011).

One may be forgiven for supposing that such debates about the nature of empathy are merely verbal—about the best way to use the term ‘empathy’. The danger of this might seem especially acute given that the term ‘empathy’ was coined as recently as the early Twentieth Century (Coplan and Goldie 2011). Of course, one is free to define the term as one pleases. I hope, however, to offer an account that combines the merits of being reasonably close to common usage of the term, of making explicit a good deal of what various theorists have wanted to say about empathy and its role in our lives, and of resisting the temptation to suppose that the term just picks out a number of phenomena whose sole uniting principle is the fact that they have been dubbed ‘empathy’. Such a broad position would deprive the notion of empathy of much of its value. On the other hand, overly narrow accounts run the risk of simply ignoring a significant part of our everyday ways of speaking of empathy. The account I propose avoids both of these vices. More positively, the fact that the concept of empathy is a relative newcomer suggests that, if it is to be retained, it must pay its way. That is, ‘empathy’ ought to pick out some phenomenon not picked out by some other well-understood term. And this in turn suggests a method: an account of empathy will ideally be one that shows it to make a distinctive contribution to our lives. Empathy makes a distinctive contribution if there is something that it and only it allows us to do. As I shall argue, empathy does make such a distinctive contribution and seeing what this is teaches us something both about our emotional lives and about the future direction of empathy research.

As the above suggests, I propose an account of what empathy is that is motivated by an answer to the question of what empathy is for. Questions about what some psychological phenomenon is for can be approached from at least two directions, an evolutionary perspective and what might be called a ‘role’ perspective. Here I follow de Vignemont and Singer,

What is empathy for? Here, it is important to distinguish between two questions: (i) why has evolution selected empathy? and (ii) what is the role of empathy now that it has emerged? The former question refers to the adaptive function of empathy, and the answer lies in studies of empathy in other species. The latter question refers to its functional role in everyday life (De Vignemont and Singer 2006, p. 439).

From an evolutionary perspective, one asks whether some phenomenon is an adaptation, (or an exaptation or a spandrel) and, if so, what it is an adaptation to. This evolutionary question will not be my focus since, with respect to empathy, it is far from clear that there is currently evidence sufficient to support one hypothesis over another (De Vignemont and Singer 2006) and, perhaps more importantly, as I will argue, there is reason to suppose that empathy is an epistemic rather than a psychological phenomenon and so not straightforwardly open to evolutionary explanation. From a role perspective, one may ask whether the phenomenon makes a distinctive contribution to our lives—a contribution that only it makes. If it does, one may ask whether that contribution is primarily cognitive or social (cf. Batson’s (2009, pp. 3–4) two questions).

I will approach the question in terms of the distinctive role that empathy currently plays in our lives, whatever its evolutionary status. That role, I will argue in Sect. 2, is primarily epistemological. In Sect. 3 I propose an account of empathy designed to serve this epistemological function. Views that identify empathy with one or other of the phenomena mentioned above are, in that respect, narrow. However, there are also broad conceptions of empathy that allow that it may take any number of these forms (Preston and De Waal 2002; Thompson 2007, Chap. 13). The view of empathy that I outline walks a line between narrow and broad definitions. On the one hand, on this account empathy is not a loosely associated group but a unitary phenomenon. On the other, many of the phenomena mentioned above may feed into empathy in a number of ways. This provides us with a helpful way of understanding the oft-mentioned relation between empathy and simulation. Whilst empathy is not the same as simulation, simulation may ground empathy in some cases. Plausibly, a further consequence of the view I propose is that empathy is not a natural kind. This has implications for how we should interpret certain experimental paradigms. They show us not about the nature of empathy itself, but about the different ways in which empathy can be achieved. I end, in Sect. 4, with the suggestion that empathy’s distinctive epistemological achievement may serve a broader social purpose, enabling what I term ‘transparent fellow feeling’.

## The epistemic role of empathy

### Sharing

The project of defining empathy in the light of an account of what it is for obviously requires us to begin with an intuitive grasp of the phenomenon. On any construal that seeks to preserve something of the contemporary common-sense notion, empathy has to do with, in some sense, sharing in or, in Deonna’s (2007) words, ‘feeling in tune with’, another person’s affective state. This much is strongly suggested by the list of candidates in the previous section (imitating, mirroring, imagining, etc.). Exactly what sense of sharing is relevant is to be determined, but just this very broad understanding is sufficient for now. The fact that empathy has to do with sharing suggests a close connection with the various phenomena that come under the heading of ‘simulation’. Indeed, a number of those candidates for empathy, mentioned above, are also often treated as ways of simulating the mental life of another (cf. Goldman 2006). Whilst there is surely a close affinity here, I will argue below that it would be wrong to suppose that we can simply treat ‘empathise’ and ‘simulate’ interchangeably. There is a distinctive role for empathy to play, even if the various claims made by simulationists turn out to be false. Empathy is a broader notion.

A number of philosophers, influenced by early Twentieth Century phenomenologists, would deny the association of empathy with sharing, at least on some interpretations of ‘share’ (Thompson 2007; Zahavi 2012). Whilst I am sympathetic with a great deal of what proponents of such a view claim, it nevertheless seems clear to me that there is a use of the term ‘empathy’, in fact its central use, according to which it involves sharing in another’s mental state. I shall, consequently, simply assume, along with the vast majority of empathy researchers, that this is the case and will not directly discuss their view here.

### Social and epistemic roles

My question, then, concerns the function of sharing in another’s affective state. Answers to this question come, by and large, in two groups: social and epistemological. First, it might be maintained that the primary, or at least a central, function of empathy is to encourage ‘pro-social’, or altruistic, behaviour (cf. Batson 2011). Certainly there is evidence that a number of the phenomena mentioned above (imitation, contagion, etc.) are apt to do this (Van Baaren et al. 2009). But it seems unlikely that empathy (or imitation, contagion, etc.) is strictly necessary for this. Simply putting someone in a good mood has similar effects (George 1991). So, whilst encouraging pro-social behaviour is perhaps one role that empathy plays, it is not its distinctive contribution. If this were the fundamental function of empathy, the concept would add little if anything to those of imitation, contagion, etc. on the one hand and altruism, on the other.

On the epistemological side, it might be suggested that the role of empathy is to provide us with knowledge of the environment or, alternatively, of what others are likely to do (Carter et al. 2009; De Vignemont and Singer 2006). If Anita resonates with Betty’s fear, then Anita may come to expect environmental danger. If she resonates with Betty’s fear and *thereby* *knows* that Betty is afraid, she may come to expect Betty to flee since, given her own fear, she is herself disposed to flee. But, once again, this is knowledge that can be gained in other ways. Whilst empathy may well play this role of distributing the cognitive burden, such a thing is in principle available otherwise, through perception, testimony or the sorts of processes to which theory theorists appeal, for example. Once more, if the provision of these varieties of knowledge were what empathy was for, the concept would not pay its way. It would remain unclear, from a role perspective, why the concept of empathy should be of much interest.

### Knowing how others feel

There is an epistemic function for empathy that is, I suggest, distinctive of it. Empathy provides us with knowledge of *how others feel* (cf. Adams 2001; Green 2007, Chap. 7; Coplan 2011). Betty may tell Anita that she is afraid. Or Anita may infer that Betty is afraid from the look on Betty’s face. Or Anita may see that Betty is afraid. Each of these can constitute, in my view, Anita’s coming to know *that* Betty is afraid. However, unless Anita empathises with Betty, Anita will not know *how* Betty feels. For that, Anita must share in Betty’s affective state. Or so I claim.

What is involved in knowing how another person feels? One might suppose that, contrary to the distinction I have drawn above, Anita’s knowing *that* Betty is afraid is sufficient for knowing *how* she feels. But this is wrong. Anita may possess a functional concept of fear, indeed she may have complete descriptive knowledge of fear, but if she has never experienced anything that shares an affective character, a way of feeling, with Anita’s fear (Damasio 2000, pp. 62–67) then she won’t know how Anita’s fear feels (Jackson 1986; Ravenscroft 1998; Brewer 2002). Rather, and generalizing for any affective psychological state $\uppsi$ (whether positive or negative), I suggest that $A$ knows how $B$ feels only if she knows that $B$ is $\uppsi$ and how it feels to be $\uppsi$. Further, $A$ knows how it feels to be $\uppsi$ if and only if $A$ knows that $\uppsi$ feels like *this*. Here ‘*this*’ is an ‘inner demonstrative’ picking out the affective character of $\uppsi$ (Perry 2003, Chap. 3). In order to secure such ‘inner demonstrative’ reference, there needs to be a conscious state of hers that $A$ can demonstrate. Thus, $A$ must be in, or perhaps have been in, some affectively matching conscious state. That is, $A$ must share in $B$’s affective state.

Never having experienced fear, or some affectively matching state, Anita has only a partial understanding of fear. Specifically, she doesn’t know how fear feels. So, whilst Anita may know *that* Betty is afraid, say on testimonial grounds, there is a certain piece of knowledge that she lacks. This is a consequence of the fact that it is only through feeling fear that Anita can become acquainted with the feel of fear, with the affective character of that emotion. I suggest, then, that the distinctive epistemological contribution of empathy is that it provides knowledge of *how* others feel. If what I have said above is correct, this seems to be something that only empathy achieves. For sharing in another’s affective state is a necessary condition of coming by this knowledge.

## What empathy is

### Empathy, conscious awareness, and knowing how others feel

What, then, is empathy? In light of the above, I propose the following:

$A$ empathises with $B$ if and only if

1. $A$ is consciously aware that $B$ is $\uppsi$,
2. A is consciously aware of what being $\uppsi$ feels like,
3. On the basis of (1) and (2), $A$ is consciously aware of how $B$ feels.

Two clarifications are in order. First, one is ‘consciously aware’ of some fact $F$ only if one is in some veridical conscious state with the representational content $F$. The state in question may be judgement, perception, or some other cognitive state with the same ‘direction of fit’. Whilst the veridicality condition does not require knowledge, it is sufficient grounds to think of this as an epistemic account of empathy, and I will occasionally use ‘knows’ where ‘veridically represents’ would be strictly appropriate. Second, the fact that one is consciously aware of some fact ‘on the basis’ of being consciously aware of some others, means that those others may be drawn upon if challenged. So, in the case of empathy, one can defend one’s claim to know how another feels by pointing out that one knows both that they are $\uppsi$ and how it feels to be $\uppsi$ ($\uppsi$, remember, is a variable ranging over affective states, either positive or negative). I do not suppose, however, that any conscious inferential process or reasoning must take place.

How might the first condition be satisfied? On the pluralistic view which, for reasons of space, I will simply assume here, it could be satisfied in any number of ways including testimony, inference, simulation and perception (cf. Goldie 1999). That is, one can come to know that $B$ is $\uppsi$ by being told that it is so; by inferring it from other things that one believes about $B$, perhaps alongside generalisations about the mind that one tacitly accepts; by simulating $B$ and attributing to her the output; or by seeing that $B$ is $\uppsi$. On this account, simulation is to be identified not with empathy *per se* but rather with one way in which the first condition might be satisfied. This is so even if one were to suppose that the empirical evidence shows that we only ever come by such knowledge by way of simulation (a claim which, in any case, is rather doubtful). For at least some of the other ways of coming to know that $B$ is $\uppsi$ are surely *possible*. On this view, then, it would be wrong to suppose that ‘simulate’ is another term for ‘empathise’. Empathy is epistemic, simulation is not. Thus, whilst there is a clear affinity between empathy and simulation, they are importantly different.

How might the second condition be satisfied? Here there is less room for variation, for the reason that in order to know what being $\uppsi$ feels like $A$ must be acquainted with the feel of $\uppsi$, for she must be able to think, ‘$\uppsi$ feels like *this*’. To be acquainted with the feel of $\uppsi$, $A$ must have experienced some state that feels that way. The most obvious way to satisfy (2), then, is if $A$ is currently $\uppsi$. Indeed, according to some ways of understanding simulationism—as involving an actual and current instantiation of a conscious state affectively matching that being simulated—the way in which $A$ comes to possess the knowledge mentioned in the first condition will typically also provide her with that mentioned in the second (cf. Goldman 2006, pp. 149–151). But this simultaneous matching of states may not be strictly necessary. I will mention three possibilities, each of which helps to further distance the concept of empathy from that of simulation.

First, it may be that $A$ can empathise with $B$’s being $\uppsi$ at $t$ although $A$ is not, at $t$, in any state that affectively matches $\uppsi$. I allow this on the condition that it is possible for $A$ to know that being $\uppsi$ feels like *this* where the inner demonstrative picks out the affective character of $\uppsi$ preserved in episodic memory (Perry 2003, pp. 58–59; Green 2007, p. 190). Allowing for this is in accordance with at least one of our ordinary ways of speaking. For it can be entirely natural for Anita to say that she empathises with Betty’s sadness since she, Anita, was in a similar situation last year but happily feels much better now. This is, I suggest, what we have in mind when we say, ‘Yep, I know that feeling’. It is important to point out, however, that in order for this possibility to meet condition (2), Anita’s episodic memory of being $\uppsi$ must be conscious, since it must be able to ground a conscious thought ‘$\uppsi$ feels like *this*’. Green, whose account of empathy is in a number of ways akin to mine, makes this point nicely,

I could save a lot of time and effort simply by calling up into conscious awareness my *memory* of how I felt [...] On the basis of that conscious awareness, I now know [or, at least, veridically represent] how you feel, not dispositionally but occurrently (Green 2007, p. 190).

Second, $A$ may empathise with $B$’s being $\uppsi$ even if $A$ has *never* been $\uppsi$. All that is required is that $A$ is, or has been, in some state that affectively matches $\uppsi$. There are a number of ways in which this condition might be met. Most obviously, the affectively matching state may be a state of the same kind, for example shame. But it may be of another kind. For example, it may be that in some instances of imaginatively representing oneself as ashamed, the affective character ‘carries over’. It is not implausible, it seems to me, that imagining performing a shameful act can in some cases feel the way shame does, to some degree at least, even though one may not satisfy the conditions on being ashamed, which may include quite sophisticated appraisals.

A final way in which $A$ may empathise with $B$’s being $\uppsi$ without ever having been $\uppsi$ has already been hinted at in the previous paragraph. It must be recognised that the affective character of a psychological state can be specified to different levels of determinacy. From a bird’s eye view, a psychological state’s affective character may just amount to pure valence: *feels good* versus *feels bad* (for a more sophisticated account see (Green 2007, Sect. 7.2)). From the snail’s eye view, affective character will be a highly nuanced super-determinate (Funkhouser 2006). Now, it is decidedly unlikely that, through emotional contagion, perspective-taking, etc. $A$ will come to be in a state with the same super-determinate affective character as $B$’s state (cf. Goldie 2011). Nevertheless, she might well come to be in some state that is similar enough (just how similar is similar enough is, plausibly, not a matter to be settled independent of context). Thus, even if $A$ is not, and has never been, in exactly the same psychological state as $B$, she may nevertheless be, or have been, in a state that affectively matches it at some level of determinacy. It is reasonable to suppose that such levels of determinacy will correspond to the *extent* to which $A$ knows, or is aware of, how $B$ feels. Again, this tallies with our linguistic practise, since we are happy to allow that one can empathise with another to a greater or lesser extent. This, I am suggesting, amounts to the extent to which one knows how they feel.

The above considerations bring out the fact that in order to empathise with $B'$s being $\uppsi$, $A$ need not be, or have been, in an affective state with the same intentional object. $A$ can empathise with $B$ even though $B$ is upset about one thing—that her child is being bullied at school—and $A$ is, or was, upset about another—that *hers* is, or was. In order that $A$ empathise with $B$ in such a case, it need not be that she has ever been upset about $B'$s child being bullied at school. This is not to say, however, that there need be no similarity of content. One might well suppose, for example, that they must be in some way analogous (in the above example, ‘my child is bullied at school’, ‘my child is bullied too’). Furthermore, insofar as $A$ is, or has been, in a state with matching affective character, there will also be matching at the level of evaluative content. At the minimum, both affective states in the above case will either represent, or be associated with a representation of, the relevant state of affairs as *bad *(Deonna and Teroni 2012). As above, the extent to which the intentional objects or evaluative contents in question must be similar is not likely to be a matter that can be decided in an entirely general way. Rather, features of the context will be crucial in determining the conditions that must be met before we will be willing to speak of empathy.

### Empathy and causation

The account on offer does not explicitly employ any causal notions. So, for example, it appears to contravene de Vignemont and Jacob’s (2012, p. 306) *causal path condition*, which would require that $A$ be in some affective state that is caused by $B$’s being $\uppsi$. I have already provided some reasons to think that $A$ need not be in an affective state at all, let alone one with any particular causal history. Nevertheless, even if those considerations are not found compelling, de Vignemont and Jacob’s formulation of the causal condition is implausible (although I am sympathetic to other elements of their view). Consider the following: $A$ and $B$ are in the waiting room of the pain-lab, ready to take part in an experiment that they know will involve them receiving a series of electric shocks. When $B$ is led off into an occluded and sound-proofed room to be given the shocks, $A$ may simulate $B$’s situation and thereby come to be in an affectively matching state, attributing that state to $B$. It is very natural to say that, in such a case, $A$ empathises with $B$ despite the fact that there is no causal path leading from the affective state of $B$ to that of $A$.

By contrast, the account that I have been articulating has the consequence that, in the envisaged scenario, $A$ empathises with $B$. I do not rule out the possibility that every case of empathising stands in some interesting causal relation to the state empathised with ($B$’s being $\uppsi$), most plausibly some restriction on _*_have a common cause*. However, if this is so, it is due to whatever causal conditions there are on the veridical representation of $B$’s being $\uppsi$, rather than to any additional condition.

### Empathy and care

It might be suggested, and has been to me on several occasions, that this account of empathy is missing a crucial ingredient. This is the condition that in order to empathise with $B$, $A$ must care about her. On the account that I have been outlining, it is possible for $A$ to empathise with $B$ whilst caring nothing for her plight. It is common to cite, for example, a torturer who, on my account, ‘empathises’ with his victim the better to inflict pain. Or we might think of Zahavi and Overgaard’s (2012) case of two angry men in a fight. Such cases do seem somewhat at odds with at least one use of ‘empathy’, according to which to empathise with someone’s suffering is, at least in part, to be moved to aid them. This is an aspect of empathy that my account leaves out.

The standard response to this sort of concern is to distinguish between empathy, on the one hand, and sympathy on the other (see, for example, Darwall 1998; Snow 2000; Nussbaum 2001, Chap. 6; Eisenberg 2007). An initial gloss on the distinction would be that whilst empathy is feeling *with* another, sympathy is feeling *for* them. Understood in this way, care clearly falls on the side of sympathy. This response is, I think, the right one. As such, I agree with Darwall when he writes that, ‘[e]mpathy can be consistent with the indifference of pure observation or even the cruelty of sadism. It all depends on why one is interested in the other’s perspective’(1998, p. 261). Furthermore, an explanation of the tendency to wrongly suppose that empathy involves care is reasonably easy to come by. First, it is notable that those philosophers, namely David Hume and Adam Smith, who were the first to articulate and put to work the concept of empathy, in fact employed the term ‘sympathy’ for this notion (Hume 1739; Smith 1759). In addition to this, there is evidence that with appropriate background conditions in place, empathy tends to lead to sympathy and, subsequently, altruistic motivation (Batson 1991). It is, then, not so surprising to find that empathy and sympathy are sometimes conflated in ordinary thought.

One recent account of empathy poses a challenge to this, however. De Vignemont and Jacob’s (2012; cf. Michael 2014) account of empathy includes the condition that the empathiser care for the empathisee. As they put it, ‘X must care about Y’s affective life’ (2012, p. 307). Far from being a mere conflation of empathy with sympathy, de Vignemont and Jacob claim that this *care condition* (CC) is empirically supported. On their view, CC is supported by the fact that ‘empathy is not the default response to one’s awareness of another’s affective state in general. In particular, empathetic pain is not the default response to one’s awareness of another’s standard pain.’ (2012, p. 307).

If correct this spells trouble for the account of empathy developed here. For adding CC to the account would quite plausibly mean that empathy, so defined, would have no *distinctive* role to play in our cognitive lives, since any such putative role could be played by empathy minus care. Fortunately for my account, however, the evidence does not support de Vignemont and Jacob’s CC. The empirical work in question is Singer et. al.’s (2006) experiment that shows that activation of neural areas associated with ‘empathic responses’ (the anterior insular/fronto-insular cortex and the anterior singulate cortex) is modulated by high-level evaluations of the other person. For example, in male subjects the neural areas in question showed significantly less activation when the other person is perceived as previously having behaved unfairly (Singer et al. 2006, p. 467). The existence of this top-down effect is what de Vignemont and Jacob have in mind when they claim that empathy is not the ‘default response’. This, they claim, supports CC.

There are a number of reasons, however, for thinking that Singer et. al.’s work does not, in fact, support CC. First, not only perceived fairness, but other contextual features, including the perceived positive benefits of the empathisee’s pain (in therapy), also modulate the mirroring responses in question (Hein and Singer 2008, p. 156). Mirroring is responsive to top–down effects and it is not obvious why *care* should be singled out. Second, in the study, perceived fairness does not seem to have the same effect for female subjects. As Singer et. al. note, ‘formal analysis revealed no significant difference for women when comparing painful trials for fair versus unfair players in empathy-related pain regions’ (Singer et al. 2006, p. 467). Given the small sample size of Singer et. al.’s study, the reliability of this data might be questioned. Nevertheless, it is far from clear that the study supports the gender neutral CC, rather than merely the possibility of top-down effects on mirroring.

The falsity of the claim that mirroring is the default response is insufficient to motivate the CC. Perhaps this is part of the reason for the fact Hein and Singer themselves explicitly distinguish empathy from sympathy (2008, p. 154). But there is a deeper reason to think that psychological or neuroscientific evidence of this sort could not in principle support the CC, or any other condition on empathy. This is that, on the account I have been offering, empathy is not a psychological phenomenon at all.

### Empathy is epistemic not psychological

According to the way of understanding empathy that I have been articulating, it is fundamentally an epistemological, not a psychological, phenomenon. That is, empathy cannot be identified with any one psychological state or process, but is more accurately described in epistemic terms, terms that themselves leave it as an open question which psychological states and processes might, in any given case, be recruited into empathy’s service. None of the psychological phenomena such as imitation, emotional contagion and perspective-taking are to be identified with empathy. Nor are any of them strictly necessary for empathy. For example, simulation is not necessary for empathy. If $A$ knows, *via* theoretical inference, that $B$ is $\uppsi$, and also, *via* memory, what it is like to be $\uppsi$, then $A$ may empathise with $B$; no simulation required. Nevertheless, as I have indicated, these various psychological phenomena can feed into empathy by grounding the veridical representations mentioned in conditions (1) and (2). On the account that I have sketched, empathy is not a process of any sort, rather it is a state in which one arrives having undergone those grounding processes, whatever they may have been. To borrow Goldman’s apt phrase, there are different ‘routes to empathy’ (Goldman 2011).

That empathy is not a psychological phenomenon gives us some reason to doubt that it is a natural kind. If A empathises, it follows that she satisfies conditions (1)–(3) but, as indicated above, conditions (1) and (2), and so (3), can be satisfied in a range of different ways. For example, one instance of empathy may be grounded in a process of simulative imagination, another in a combination of testimony and memory. Thus, even if we suppose such psychological states as imagination, memory, and, more controversially, testimonial knowledge, each to be natural kinds, the psychological states that an empathising subject may be in are so disparate that there is little to recommend the view that empathy itself is a kind. Accordingly, it would be surprising if empirically grounded judgements about empathy were projectable. If $K$ is a natural kind, some empirically grounded judgements about a sample of $K$s—those making reference to the features that distinguish $K$ from other kinds—may be reliably, although perhaps not exceptionlessly, projected to the whole category (cf. Goodman 1955, Chap. 4; Quine 1969). Whilst there are various conceptions of natural kinds (see, for example, Bird and Tobin 2012), all are associated with the idea that the surface features of members of a kind are explained by ‘underlying’ (microstructural, evolutionary, etc.) similarities, and that these underlying similarities may serve as the basis for empirically grounded inductive generalisations.

A familiar example would be the discovery that the atomic number of gold is 79. This discovery, of necessity limited to a sample, may be projected to all samples of gold. But nothing similar can be said for empathy. There is little reason to suppose that empirical discoveries about some sample instances of empathy may be projected to all instances, since the wide variety of possible cases of empathy display little or no underlying (microstructural, evolutionary, etc.) similarity. They do not tell us about the nature of empathy but, more plausibly, about the nature of those psychological states that do the work in satisfying conditions (1) and (2).

Consider, for example, the experiment mentioned above, Singer et. al.’s (2006) experiment showing that activation of neural areas associated with what they call ‘empathic responses’ is modulated by high-level evaluations of the other person. Of course, this only tells us something about empathy itself if we have reason to believe that the anterior insular/fronto-insular cortex and the anterior singulate cortex are associated with empathy *per se*. What, though, are Singer and colleagues referring to with the term ‘empathic responses’? This is what they tell us,

Empathy enables us to share the emotion, pain and sensation of others. The perception-action model of empathy states that the observation or imagination of another person in a particular emotional state automatically activates a representation of that state in the observer [...] our ability to empathize relies on neuronal systems that underpin our own bodily and emotional states. (Singer et al. 2006, p. 466)

What Singer and colleagues have in mind, then, is what I have been calling ‘mirroring’ or ‘resonance’. But this, I have been arguing, is not identical to empathy. Rather, mirroring provides one possible way in which empathy might be achieved. As a result, at best, what this experimental paradigm shows is that mirroring, a phenomenon which may underpin some instances of empathy, can be modulated by high-level evaluations. This cannot be projected onto the full spectrum of instances of empathy. It tells us nothing, for example, of cases in which A empathises with B’s distress through being reliably informed of her plight and knowing how it must feel through her presently being in a sufficiently similar situation.

If empathy is not a natural kind, there is no reason to suppose that the results of experiments such as that of Singer and colleagues are projectable and so they cannot, in principle, support conditions such as de Vignemont and Jacob’s CC. Experimental paradigms such as that of Singer and colleagues tell us about mirroring, not about empathy *per se*. Analogous remarks apply to a number of other experimental paradigms, for example Avenanti and colleagues’ experiments on pain mirroring, and their resulting claim that, ‘empathy for pain may rely not only on affective-motivational but also on fine-grained somatic representations’ (Avenanti et al. 2005, p. 958). Again, what the experiments show us is something about mirroring. There is no reason to suppose that such claim can be projected onto every instance of empathy with another’s pain.

To forestall possible misunderstanding, I am not suggesting that there is anything wrong with these experimental paradigms or others like them. Not at all. My point is simply that these paradigms do not show us something about the nature of empathy itself, but rather of the sorts of psychological processes that are plausibly thought to underlie some, but not all, instances of it. Nor does the complaint apply equally to all empirically grounded claims about empathy. For example, the problematic cases above stand in contrast to others, such as Wicker and colleagues’ (2003) disgust paradigm and the thought, in Goldman’s words, that it shows that, ‘observing a disgust expressive face produces mental mimicry, or empathy’ (2011, p. 35). Here there is no illicit projection, just the claim that empathy may be provoked in a certain way. This makes no claim about the nature of empathy, and is entirely consistent with the approach to empathy that I have been endorsing.

## A social role for empathy

I have claimed that the function of empathy is to provide knowledge of *how* others feel. However, I would also like to suggest that, by way of this distinctive epistemological achievement, empathy may serve a broader social function. As Adam Smith recognised—in fact he based a significant part of his moral theory on it—we place a high value on empathy (Smith 1759). As he puts it, ‘nothing pleases us more than to observe in other men a fellow-feeling with all the emotions of our own breast’ (1759, I.i.2.2). His term ‘fellow-feeling’ is highly appropriate here. One of the things we value is feeling *with* others (Tomasello 2008, pp. 210–212). Whilst merely feeling as another does is of value, I suggest that we place a higher value on knowing that the other feels the same, and knowing that the other knows that one feels the same, etc. We have here something of the structure of common knowledge (Lewis 1969, Chap. 2; Schiffer 1972, pp. 30–42). We might call this complex state of affairs ‘transparent fellow-feeling’.

I have claimed that it is only through empathy that a person can know how another feels. Thus, since transparent fellow-feeling involves knowing how another feels, it follows that empathy is necessary for transparent fellow-feeling. One might be tempted to suppose that, contrary to this, transparent fellow-feeling could occur in the absence of knowing *how* the other feels, just getting along with an awareness of *what* they feel. But this would be a mistake. For, if $A$ fellow-feels with $B$ and $A$ knows *what* $B$ feels then $A$ will thereby know *how* B feels. Transparent fellow-feeling entails knowing *how*. Empathy, then, is necessary for transparent fellow-feeling.

It may be, however, that only some instances of transparent fellow-feeling possess the sort of value to which I have alluded. Cases of transparent fellow-feeling between sadists come to mind, for example. Perhaps more interesting would be cases of alienation, in which one party does not reflectively endorse their own emotional state—for example if I share a racist joke, but am at the same time appalled at my finding it funny. These sorts of cases aside, Adam Smith’s idea contains an important truth—that we do indeed find value in transparent fellow-feeling. Of course it is no doubt the case that knowing how others feel is important for pro-social behaviour. But it might also be that we think of transparent fellow-feeling as an end in itself. If so, then empathy serves a social function that is itself independent of any further pro-social effects it may have. We value feeling with others.

## Conclusion

The concept of empathy is contested and in offering an account of it some stipulation is perhaps inevitable. I hope, nevertheless to have presented a view that has the benefits of being reasonably true to both ordinary ways of speaking about empathy and also the various claims that have been made about it by a wide range of theorists in a variety of disciplines. I have suggested that, whilst related, ‘empathy’ should not be considered as another term for simulation. Empathy has its own role in our lives: it alone allows us to know how others feel. I have also suggested that, on the account I have offered, empathy is not a natural kind and so certain experimental paradigms tell us not about the nature of empathy but about certain ways in which empathy can be achieved. I add the speculation that, thought of in this way, empathy may also play a distinctive social role, enabling the valuable state of ‘transparent fellow-feeling’.

## References

[CR1] Adams FR (2001). Empathy, neural imaging and the theory versus simulation debate. Mind and Language.

[CR2] Avenanti A, Bueti D, Galati G, Aglioti SM (2005). Transcranial magnetic stimulation highlights the sensorimotor side of empathy for pain. Nature Neuroscience.

[CR3] Batson CD (1991). The altruism question: Toward a social-psychological answer.

[CR4] Batson CD, Decety J, Lckes W (2009). These things called empathy: Eight related but distinct phenomena. The social neuroscience of empathy.

[CR5] Batson CD (2011). Altruism in humans.

[CR6] Batson CD, Early S, Salvarani G (1997). Perspective taking: Imagining how another feels versus imaging how you would feel. Personality and Social Psychology Bulletin.

[CR7] Bird A, Tobin E, Zalta EN (2012). Natural kinds. The stanford encyclopedia of philosophy.

[CR8] Brewer B, Goldie Peter (2002). Emotion and other minds. Understanding emotions: Mind and morals.

[CR9] Carter CS, Harris J, Porges SW, Decety J, Ickes W (2009). Neural and evolutionary perspectives on empathy. The social neuroscience of empathy.

[CR10] Coplan A, Coplan A, Goldie P (2011). Understanding empathy: Its features and effects. Empathy: Philosophical and psychological perspectives.

[CR11] Coplan A, Goldie P (2011). Empathy: Philosophical and psychological perspectives.

[CR12] Damasio AR (2000). The feeling of what happens: Body, emotion and the making of consciousness.

[CR13] Darwall S (1998). Empathy, sympathy, care. Philosophical Studies.

[CR14] Deonna J (2007). The structure of empathy. Journal of Moral Philosophy.

[CR15] Deonna J, Teroni F (2012). The emotions: A philosophical introduction.

[CR16] De Vignemont F, Jacob P (2012). What is it like to feel another’s pain?. Philosophy of Science.

[CR17] De Vignemont F, Singer T (2006). The empathic brain: How, when and why?. Trends in Cognitive Sciences.

[CR18] Eisenberg N, Bock G, Goode J (2007). Empathy-related responding and prosocial behavior. Empathy and fairness.

[CR19] Funkhouser E (2006). The determinable–determinate relation. Noûs.

[CR20] George JM (1991). State or trait: Effects of positive mood on prosocial behaviors at work. Journal of Applied Psychology.

[CR21] Goldie P (1999). How we think of others. Emotions’. Mind and Language.

[CR22] Goldie P, Coplan A, Goldie P (2011). Anti-empathy. Empathy: Philosophical and psychological perspectives.

[CR23] Goldman AI (2006). Simulating minds: The philosophy, psychology, and neuroscience of mindreading.

[CR24] Goldman AI, Coplan A, Goldie P (2011). Two routes to empathy: Insights from cognitive neuroscience. Empathy: Philosophical and psychological perspectives.

[CR25] Goodman N (1955). Fact, fiction, and forecast.

[CR26] Green M (2007). Self-expression.

[CR27] Hatfield E, Rapson RL, Le Y-CL, Decety J, Ickes W (2009). Emotional contagion and empathy. The social neuroscience of empathy.

[CR28] Hein G, Singer T (2008). I feel how you feel but not always: The empathic brain and its modulation. Current Opinion in Neurobiology.

[CR29] Hume, D. (1739). *A Treatise of Human Nature* (L. A. Selby-Bigge & P. H. Nidditch, Eds.). Second Edition. Oxford: Clarendon Press.

[CR30] Jackson F (1986). What Mary didn’t know. The Journal of Philosophy.

[CR31] Jacob, P. The disunity of vicarious experiences (Unpublished).

[CR32] Lewis D (1969). Convention: A philosophical study.

[CR33] Michael J (2014). Towards a consensus about the role of empathy in interpersonal understanding. Topoi.

[CR34] Nussbaum M (2001). Upheavals of thought: The intelligence of emotions.

[CR35] Perry J (2003). Knowledge, possibility, and consciousness.

[CR36] Preston SD, De Waal FBM (2002). Empathy: Its ultimate and proximate bases. Behavioral and Brain Sciences.

[CR37] Quine WV (1969). Natural kinds. Ontological relativity and other essays.

[CR38] Rapson RL, Hatfield E, Cacioppo JT (1994). Emotional contagion.

[CR39] Ravenscroft I (1998). What is it like to be someone else? Simulation and empathy. Ratio.

[CR40] Schiffer S (1972). Meaning.

[CR41] Singer T, Seymour B, O’Doherty JP, Stephan KE, Dolan RJ, Frith CD (2006). Empathic neural responses are modulated by the perceived fairness of others. Nature.

[CR42] Smith, A. (1759). *The theory of moral sentiments* (D. D. Raphael & A. L. Macfie, Eds.). Oxford: Oxford University Press, 1976.

[CR43] Snow NE (2000). Empathy. American Philosophical Quarterly.

[CR44] Thompson E (2007). Mind in life: Biology, phenomenology, and the sciences of mind.

[CR45] Tomasello M (2008). Origins of human communication.

[CR46] Van Baaren R, Decety J, Dijksterhuis A, Van der Leij A, Van Leeuwen ML, Decety J, Ickes W (2009). Being imitated: Consequences of nonconsciously showing empathy. The social neuroscience of empathy.

[CR47] Wicker B, Keysers C, Plailly J, Royet J-P, Gallese V, Rizzolatti G (2003). Both of us disgusted in my insula: The common neural basis of seeing and feeling disgust. Neuron.

[CR48] Zahavi D (2012). Empathy and direct social perception: A phenomenological proposal. Review of Philosophy and Psychology.

[CR49] Zahavi, D., & Overgaard, S. (2012). Empathy without isomorphism: A phenomenological account. In J. Decety (Ed.), *Empathy from bench to bedside* (pp. 3–20). Cambridge, MA: MIT Press.

